# A CRISPR-Cas9–integrase complex generates precise DNA fragments for genome integration

**DOI:** 10.1093/nar/gkab123

**Published:** 2021-03-08

**Authors:** Shrutee Jakhanwal, Brady F Cress, Pascal Maguin, Marco J Lobba, Luciano A Marraffini, Jennifer A Doudna

**Affiliations:** Department of Molecular and Cell Biology, University of California, Berkeley, Berkeley, CA 94720, USA; California Institute for Quantitative Biosciences, University of California, Berkeley, Berkeley, CA 94720, USA; Innovative Genomics Institute, University of California, Berkeley, Berkeley, CA 94720, USA; Department of Molecular and Cell Biology, University of California, Berkeley, Berkeley, CA 94720, USA; California Institute for Quantitative Biosciences, University of California, Berkeley, Berkeley, CA 94720, USA; Innovative Genomics Institute, University of California, Berkeley, Berkeley, CA 94720, USA; Laboratory of Bacteriology, The Rockefeller University, New York, NY 10065, USA; Department of Molecular and Cell Biology, University of California, Berkeley, Berkeley, CA 94720, USA; California Institute for Quantitative Biosciences, University of California, Berkeley, Berkeley, CA 94720, USA; Department of Chemistry, University of California, Berkeley, Berkeley, CA 94720, USA; Laboratory of Bacteriology, The Rockefeller University, New York, NY 10065, USA; Department of Molecular and Cell Biology, University of California, Berkeley, Berkeley, CA 94720, USA; California Institute for Quantitative Biosciences, University of California, Berkeley, Berkeley, CA 94720, USA; Innovative Genomics Institute, University of California, Berkeley, Berkeley, CA 94720, USA; Department of Chemistry, University of California, Berkeley, Berkeley, CA 94720, USA; Howard Hughes Medical Institute, University of California, Berkeley, Berkeley, CA 94720, USA; Molecular Biophysics and Integrated Bioimaging Division, Lawrence Berkeley National Laboratory, Berkeley, CA 94720, USA; Gladstone Institute of Data Science and Biotechnology, Gladstone Institutes, San Francisco, CA 94158, USA

## Abstract

CRISPR-Cas9 is an RNA-guided DNA endonuclease involved in bacterial adaptive immunity and widely repurposed for genome editing in human cells, animals and plants. In bacteria, RNA molecules that guide Cas9′s activity derive from foreign DNA fragments that are captured and integrated into the host CRISPR genomic locus by the Cas1-Cas2 CRISPR integrase. How cells generate the specific lengths of DNA required for integrase capture is a central unanswered question of type II-A CRISPR-based adaptive immunity. Here, we show that an integrase supercomplex comprising guide RNA and the proteins Cas1, Cas2, Csn2 and Cas9 generates precisely trimmed 30-base pair DNA molecules required for genome integration. The HNH active site of Cas9 catalyzes exonucleolytic DNA trimming by a mechanism that is independent of the guide RNA sequence. These results show that Cas9 possesses a distinct catalytic capacity for generating immunological memory in prokaryotes.

## INTRODUCTION

CRISPR-Cas (**C**lustered **R**egularly **I**nterspaced **S**hort **P**alindromic **R**epeats-CRISPR-associated) bacterial adaptive immune systems capture and store viral sequences in the genome for expression as guide RNAs that direct Cas proteins to disrupt viral infection (1). In type II CRISPR-Cas systems, Cas9 is the RNA-guided DNA endonuclease that targets and cuts double-stranded DNA bearing complementarity to the guide RNA sequence (2,3). Cas1 and Cas2 are phylogenetically conserved proteins that integrate virally derived DNA spacers into the CRISPR genomic sequence array while preserving the genomic integrity of the host (4–8). Although the enzymatic functions of Cas9-guide RNA and the Cas1-Cas2 integrase were defined independently, several lines of evidence suggested that in bacteria, these components function together to both acquire and deploy new viral sequences in the CRISPR immune pathway (9,10).

First, Cas9 helps select spacer sequences with a short PAM sequence motif at one end, preventing autoimmunity in host cells (11). Second, Cas9 can assemble with Cas1, Cas2 and a third protein, Csn2, to form a supramolecular complex, within which its function is unclear (9,12,13). Third, although a Cas9 mutation enhanced DNA spacer acquisition *in vivo*, the mechanism for this observation has remained elusive due in part to the dynamic nature of the Cas9-integrase supercomplex (14). Structural studies showed that a subcomplex including Cas1, Cas2 and Csn2 binds to DNA spacer molecules of the correct 30-base pair length for CRISPR array integration (13), but the absence of Cas9 in this complex left the question of its role in the acquisition process unanswered. Finally, neither the Cas1-Cas2-Csn2 spacer capture complex structure nor the mechanism of site-specific DNA integration catalyzed by Cas1-Cas2 (4,10,15) can explain the generation of prespacer DNA integration substrates, leading us to hypothesize a role for Cas9 in this process.

Using a combination of cell-based experiments and biochemical reconstitution, we show here that a guide RNA-Cas1-Cas2-Csn2-Cas9 integrase supercomplex processes DNA substrates to the correct length for integration *in vitro*, and that the Cas9 component provides this enzymatic activity by a mechanism that is independent of RNA-guided DNA binding. *In vivo* experiments show that the nuclease activity of Cas9 is dispensable for acquiring canonical sized substrates, indicating that cellular nucleases other than Cas9 can also perform this DNA trimming activity. Taken together, our work shows how the guide RNA-independent exonucleolytic activity of Cas9 helps to generate canonical sized substrates for integration by the Cas1-Cas2 integrase and provides a better understanding for the role of Cas9 in the adaptation pathway.

## MATERIALS AND METHODS

### Bacterial strains and growth conditions

*Staphylococcus aureus* RN4220 (16) was cultured in heart infusion (HI) or broth heart infusion (BHI) at 37°C. For plasmid maintenance, 10 μg/ml chloramphenicol was added to the media when applicable. For all the infection experiments, phage ΦNM4γ4 was used (9).

### Plasmid construction

Construction of the plasmids containing the *Streptococcus pyogenes* Type IIA CRISPR-Cas system with a single repeat and either wild-type Cas9 (pGG32) or dCas9 (pRH227) on the pC194 backbone (17) were described in a previous publication (9). Versions of pGG32 containing D10A Cas9 (pPM246) and H840A Cas9 (pPM247) were constructed from two-piece Gibson assemblies (18) by amplifying pGG32 with the primer pair H064-H295 and using either pRH202 or pRH203 amplified with H061-H296, respectively. pRH202 and pRH203 were also assembled using 2-piece Gibson reactions by amplifying pRH087 (9) with primer pairs L431-B337 and B295-B338 for pRH202 and with L431-B339 and B295-B340 for pRH203. Plasmids containing the *S. pyogenes* Type IIA (Δ*Cas1*, Δ*Cas2* and Δ*Csn2*) with ΦNM4γ4 targeting spacers of different lengths were built by inserting the spacers in pBD184 (19) using BsaI cloning. The spacers were made and inserted in the plasmids by annealing the following primer pairs and ligating them in BsaI digested pDB184: PM1206-PM1207 (pPM235–30bp), PM1266-PM67 (pPM235–35bp), PM1268-PM1269 (pPM235–40bp) and PM1270-PM1271(pPM235–45bp). All the constructed plasmids were electroporated in *S. aureus* RN4220 as previously described elsewhere (19). A list of all oligonucleotide sequences used for PCR and molecular cloning in the *S. aureus* (*in vivo*) experiments are mentioned in Supplementary Table S1.

### Protein expression and purification

The co-expression of the supercomplex was done using a construct containing His_10_-Cas9-Cas1-Cas2-Csn2 as reported earlier (9). The plasmid containing the co-expression vector was expressed in *Escherichia coli* BL21 Rosetta (DE3) cells. Cultures were induced for 16 h with isopropyl β-D-1-thiogalactopyranoside (IPTG) and the cells were lysed by sonication. The cell lysate was first purified by Ni-NTA affinity chromatography. The eluates were dialyzed and further subjected to ion-exchange chromatography by using Heparin columns. For ion-exchange chromatography, elution was performed using a concentration gradient with Buffer A (20 mM HEPES, 1000 mM NaCl, 1 mM TCEP, 1 mM EDTA, 5% glycerol at a pH of 7.4) and Buffer B (20 mM HEPES, 0 mM NaCl, 1 mM TCEP, 1 mM EDTA, 5% glycerol at a pH of 7.4). For binding and molecular weight measurements, the elution peak from the Heparin column containing all four proteins was mixed with a spacer and sgRNA of choice and incubated at 4$^\circ$C for 2 h before injecting into a Superdex 200 10/300 increase column. The buffer composition was 20 mM HEPES, 300 mM NaCl, 1 mM TCEP, 1 mM EDTA, 5% glycerol at a pH of 7.4. Variants of the supercomplex containing either Cas9 nickase (D10A or H840A) or a dCas9 (D10A and H840A) were created by introducing point mutations into the parent construct by golden-gate cloning and were purified using the protocol mentioned above. Cas1 and Cas2 proteins were purified using the same protocol as reported earlier (15).

### DNA and sgRNA preparation

All DNA oligonucleotides used in this study were synthesized by integrated DNA technologies. Prespacers were prepared by annealing the complementary strands in hybridization buffer (20 mM HEPES, 100 mM NaCl, 5 mM MgCl_2_, pH 7.4) and heating at 95°C for 5 min followed by slow cooling to room temperature. A list of all different prespacers used in this study have been shown in Supplementary Tables S2, S4, S5 and S6. The sgRNAs used in this study were synthesized by integrated DNA technologies. The sgRNA sequences used for all *in vitro* processing assays and for selection of full-site integration products are:

Comp sgRNA:

UCUUCGAAGACCGUUUCUUUGUUUUAGAGCUAUGCUGUUUUGAAAAAAACAGCAUAGCAAGUUAAAAUAAGGCUAGUCCGUUAUCAACUUGAAAAAGUGGCACCGAGUCGGUGCUUCG

Non-comp sgRNA:

GCUUAGAUAGUCGAUAGCAUGUUUUAGAGCUAUGCUGUUUUGAAAAAAACAGCAUAGCAAGUUAAAAUAAGGCUAGUCCGUUAUCAACUUGAAAAAGUGGCACCGAGUCGGUGCUUCG

sgRNA scaffold:

GUUUUAGAGCUAUGCUGUUUUGAAAAAAACAGCAUAGCAAGUUAAAAUAAGGCUAGUCCGUUAUCAACUUGAAAAAGUGGCACCGAGUCGGUGCUUCG

### SEC-DALLS measurement

Stoichiometry determination of the supercomplex was performed using size-exclusion chromatography coupled to dual-angle laser light scattering. Supercomplex was first purified using Ni-NTA affinity chromatography followed by ion-exchange chromatography on a Heparin column. The purified fraction was complexed with a pre-spacer DNA and an sgRNA having sequence complementarity to the pre-spacer DNA being added. The complex was then injected into Superdex 200 10/300 Increase coupled to an Agilent 1260 Infinity Multi-Detector system. The buffer composition was 20 mM HEPES, 300 mM NaCl, 1 mM TCEP, 1 mM EDTA, 5% glycerol at a pH of 7.4. A 658 nm laser was used to perform light scattering. UV (280 nm), UV (260 nm) and light scattering signals at 90° were collected. The eluting fractions were analyzed to assess the molecular weights of the components. Bio-SEC software (Agilent) was used for data analysis and molecular mass calculations.

### *In vitro* prespacer processing assay

For prespacer processing experiments, Cas1-Cas2 integrase or the supercomplex with a complementary sgRNA/non-complementary sgRNA/sgRNA scaffold were incubated together at a concentration of 0.5 μM on ice for 1 h. The respective protein mixtures (Cas1 and Cas2) or the ribonucleoprotein complexes (supercomplex or Cas9) was incubated with 1 μM annealed prespacer substrates for 60 min at 37°C in processing buffer (HEPES 20 mM, KCl 200 mM, DMSO 10%, MgCl_2_ 5mM, pH 7.4). The reactions were quenched with 25 mM EDTA and Proteinase K. Loading buffer containing bromophenol blue (BPB) was added to the reactions and heated at 80$^\circ$C for 10 min. The samples were then separated on 15% Urea-PAGE. Prespacer substrates used in all the processing reactions were unlabeled and the bands were visualized by post-staining the gels with SYBR gold. All the end-point experiments were performed at a 60-min time-point whereas the kinetic measurements were performed at time-points of 5, 10, 20, 40, 60 and 120 min. Quantitation of the processed products was performed from three replicates using Image lab software. Linear regression was used to determine the processing rates using Graph Pad Prism, 8.0.

### Selection for full-site integration products

Prespacer substrates were designed to facilitate Golden Gate-mediated insertion of a chloramphenicol selection cassette into pCRISPR (containing an Ampicillin cassette) only upon full-site integration of the prespacer by Cas1-Cas2 integrase or the supercomplex. Specifically, two abutted and inverted BbsI restriction sites were encoded in the center of each prespacer substrate, with overhangs designed to be complementary to the BbsI-generated overhangs at the edges of the chloramphenicol selection cassette. In total, the BbsI recognition and cleavage sites occupied the central 24 bp of each prespacer. *In vitro* integration reactions were performed with the designed prespacers as described in the section above. The reactions were quenched by the addition of 25 mM EDTA. The products of the integration reactions were purified using a DNA Clean and Concentrator 5 kit (Zymo Research) and eluted with 6 μl water. The purified products were subjected to a gap-filling reaction (20 μl total, 37 C for 30 min) in which 3′ ends were extended by a non-displacing DNA polymerase and nicks were ligated: 6 μl purified acquisition reaction, 2 μl Taq DNA ligase (80 U, NEB), 1 μl T4 DNA Polymerase (1 U, NEB), dNTP Solution Mix (1 mM each, NEB), 2 μl 10x Taq DNA Ligase buffer (NEB), 6.5 μl water. Gap-filling reactions were again purified using the Zymo Research kit as described above. A Golden Gate-compatible chloramphenicol cassette was generated by PCR using a forward primer:

GGCCGAAGACGCAGATCTTATATCGTATGGGGCTGACTTCAGGTTGATCGGGCACGTAAGAGGTTCC

and a reverse primer:

GGCCGAAGACGCGAAACTTATATCGTATGGGGCTGACTTCAGGACCAATAAAAAACGCCCGGC

encoding BbsI sites, and this amplicon was purified with the E.Z.N.A Cycle Pure Kit (Omega Biotek). The purified gap-filled products and chloramphenicol selection cassette were subjected to Golden Gate cloning using a standard BbsI assembly protocol.

Golden Gate reactions were purified using the Clean and Concentrate 5 kit as described above, eluted in 6 μl H_2_O, and 3 μl was electroporated into 25 μl *E. coli* strain E. cloni 10G Elite electrocompetent cells (Lucigen). Electroporated cells were recovered in 975 μl pre-warmed Lucigen recovery medium for 1 h at 37 C, serially diluted in LB media, spot-plated (5 μl per spot) on LB agar containing 100 μg/ml carbenicillin and 25 μg/ml chloramphenicol and incubated overnight at 37 C. Individual colonies were picked into 1 ml TB medium containing carbenicillin and chloramphenicol in 96 deep well blocks (Costar) and grown in a shaking incubator (Multitron) overnight at 37°C and 750 rpm. Plasmids were purified and Sanger sequenced with primers binding inside the chloramphenicol resistance cassette and oriented outward toward the flanking CRISPR array:

GGCCCGTCTCACTTTCATTGCCATACGAAATTCCGGATGAGC

and GGCCCGTCTCGAAAGACGGTGAGCTGGTGATATGG.

For each colony, the forward and reverse Sanger sequencing reads were concatenated and annotated using a reference feature database derived from known features on pCRISPR. Colonies were excluded from analysis if Sanger sequencing traces were low quality or uninterpretable; if the spacer was integrated at a site other than the edge of a direct repeat; if the integrated spacer was flanked by nonconsecutive spacers from the original CRISPR array; or, in rare circumstances, if mutations or indels in the prespacer ends were observed (likely stemming from oligonucleotide synthesis errors).

### Infection growth curves

Overnight cultures were diluted 1:100 in 5 ml of BHI with 5 mM CaCl_2_ and 10 μg/ml of chloramphenicol. The cultures were then grown for 1 h and 15 min and normalized to OD_600_ = 0.3. About 150 μl of each culture was added to three wells of a 96-well plate. When appropriate, bacteriophage was added directly to the well at a MOI of 1. Bacterial growth was monitored over time in a microplate reader (TECAN Infinite 200 PRO) by recording the OD_600_ of each well every 10 min.

### Plaque formation assay

About 100 μl of an overnight culture mixed with melted 50% heart infusion agar with 5 mM CaCl_2_ and 10 μg/ml of chloramphenicol was plated on BHI plates. About 3 μl of 10-fold serial dilutions of phage were plated on the lawn of cells once the agar had solidified. The next day, photos of the plates were taken using a plate imager (FluorChem HD2).

### Spacer acquisition in *S. aureus*

Spacer acquisition was performed as previously described (20) with slight modifications. Briefly, overnight cultures from single colonies were diluted 1:100 in BHI with 5 mM CaCl_2_ and grown for 1 h and 15 min. Then, the optical density (OD_600_) was recorded, and all the cultures were diluted to OD_600_ = 0.3. The cultures were infected with ΦNM4γ4 at a MOI of 100 for 30 min. Next, the cells were pelleted at 10 000 RPM for 3 min and flash frozen in liquid nitrogen. The frozen pellets were stored at -80°C until plasmid extraction for next-generation sequencing.

### PCR amplification of CRISPR loci for next-generation sequencing

Plasmid from frozen RN4220 pellets were obtained using a modified QIAprep Spin Miniprep Kit protocol described elsewhere (21). PCR amplifications of the CRISPR loci were performed as previously described with slight modifications (20). Briefly, 250 ng of DNA was used for amplification with primer PM168 and a cocktail of three primers: PM375, PM376 and PM377. For each sample, a modified PM169 primer containing five random nucleotides and a unique 3–5 bp barcode at its 5′-end was used to track each sample in the sequencing data. The PCR products were analyzed on a 2% agarose gel and locations corresponding for expanded CRISPR loci were extracted. Samples preparation for high-throughput sequencing was performed exactly as previously described (20).

### High-throughput sequencing data analysis

Using a custom Python script, the spacer sequences aligning perfectly to ΦNM4γ4′s genome was extracted and the number of reads and the length for each spacer were recorded. The number of reads for each spacer sequence was normalized to account for PCR biased as previously described (21). For each sample, the number of unique spacer sequences with the same length was calculated and graphed as a percentage of all unique spacer sequences in that sample.

## RESULTS

### Demonstration of spacer integration and function *in vivo*

**Figure 1.**
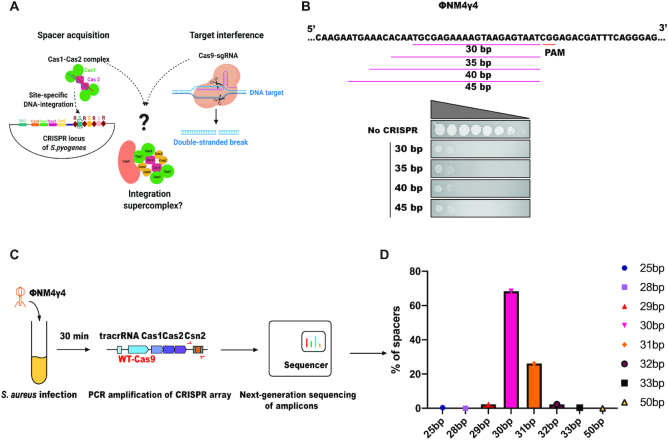
CRISPR spacer integration and spacer function are distinct *in vivo*. (**A**) Schematic representing the intersection of the integration and interference machineries for the formation of a Cas9-integration supercomplex. (**B**) Lengths of different spacers designed to target the protospacer sequence in the genome of ΦNM4γ4 phage. Ten-fold serial dilutions of ΦNM4γ4 plated on lawns of *S. aureus* RN4220 harboring an empty plasmid or a plasmid containing *S. pyogenes* type II-A CRISPR-Cas system (ΔCas1, ΔCas2, and ΔCsn2) with spacers of different lengths. (**C**) Schematic of the experimental design for studying the length of spacers acquired upon phage infection. *Staphylococcus aureus* RN4220 cultures harboring a plasmid containing the *Streptococcus pyogenes* type II-A CRISPR-Cas system (with a single repeat and wild-type (WT)- Cas9) was infected with phage ΦNM4γ4. Thirty minutes post-infection, the plasmids were isolated and the CRISPR loci were amplified by PCR. Phage-derived spacers were analyzed through next-generation sequencing of the PCR amplicons. (**D**) Deep sequencing analysis of spacer lengths acquired 30 min after phage infection of *S. aureus* RN4220.

The physical association between the type II-A Cas1, Cas2, Csn2 and Cas9 proteins has previously been established (9,12,13) but the role of this complex in the adaptation pathway has remained unclear. Using the co-purified complex of *S. pyogenes* Cas1, Cas2, Csn2, Cas9 complexed with a guide RNA, we aimed to determine its role in the acquisition of new spacers into the CRISPR array (Figure 1A and Supplementary Figure S1). CRISPR arrays acquire spacers of a strictly defined length adjacent to the leader-proximal repeat *in vivo* (15,22). However, under *in vitro* conditions, CRISPR integrases have been shown to acquire spacers of lengths spanning a wider range and at more promiscuous integration sites (4). To determine whether CRISPR function requires spacers of a specific length in bacteria, we first tested whether the physiological 30-bp spacer length is critical to establish phage immunity in the type II-A CRISPR-Cas system. Plasmids encoding spacers 30–45 base pairs in length and complementary to sequences adjacent to a protospacer-adjacent motif (PAM) sequence in ΦNM4γ4 phage (9) (Figure 1B) were introduced into *Staphylococcus aureus* RN4220 cultures (a strain lacking natural CRISPR-Cas loci) (16) and harboring the *S. pyogenes* CRISPR-Cas9 gene (9). Results of a plaque-formation assay after ΦNM4γ4 phage challenge (Figure 1B) showed no difference in protection from phage infection with the different spacers, indicating that nonphysiological spacer length does not compromise CRISPR-based immunity (Supplementary Figure S2). This is, however, not surprising since Cas9 only protects ∼20 nucleotides of the spacer from degradation by cellular RNases. The additional sequence encoded by the longer spacers is therefore unlikely to affect the Cas9-crRNA-tracrRNA complex involved in target interference. We next tested the length of spacers acquired *in vivo* by conducting adaptation experiments in which *S. aureus* RN4220 cultures containing a *S. pyogenes* type II-A CRISPR-Cas system (lacking the natural CRISPR array from *S. pyogenes* SF370 and harboring a single repeat) were challenged with ΦNM4γ4 phage (Figure 1C). Deep sequencing of the acquired spacers showed that a majority of those spacers were 30–31bp in length (Figure 1D). These results showed that although longer spacers are not detrimental to the system, the CRISPR integrase acquires precise-length spacers *in vivo* (4,8,15), implying that the integration machinery itself, or its interactions with cellular factors, generate integration substrates that are optimal for integrase chemistry.

### Cas9-integrase supercomplex trims integration substrates to a canonical size *in vitro*

**Figure 2.**
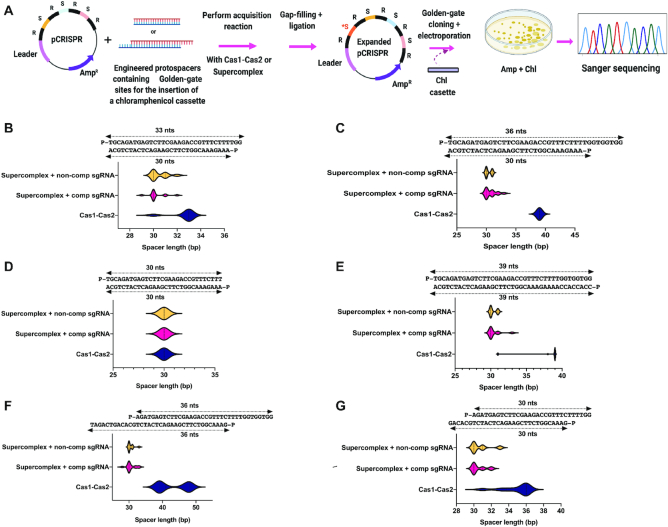
Cas9-integrase supercomplex generates physiologically relevant substrates –CRISPR integration. (**A**) Schematic of a novel screen developed for the selection of full-site integration products from the type II-A CRISPR-Cas acquisition machinery. R: Repeats; S: Spacers; *S: New spacer (**B–G**), distribution of spacer lengths for different kinds of prespacers upon full-site integration by Cas1-Cas2 integrase (blue), supercomplex + complementary sgRNA (pink) and supercomplex + non-complementary sgRNA (yellow). Sequence of substrates and population size for each given prespacer and complexes has been listed in Supplementary Tables S2 and S3, respectively.

Previous studies using *in vitro* assays from the type II-A system focused on the final step of the integration pathway (15,23) without addressing the mechanisms of integration substrate production. To test whether the Cas9-integrase supercomplex (including Cas1, Cas2, Csn2, Cas9 and guide RNA; hereafter referred to as the supercomplex (SC)) is capable of DNA fragment processing and accurate integration, we devised an acquisition assay in which products formed during *in vitro* plasmid integration reactions are selected following transformation into *E. coli* (Figure 2A). Acquisition reactions were conducted using DNA fragments of different lengths and containing internal restriction sites to accommodate subsequent insertion of a selectable marker. A chloramphenicol resistance cassette cloned into newly inserted spacers enabled integration product selection by bacterial transformation and outgrowth in presence of chloramphenicol and ampicillin (Figure 2A). CRISPR arrays from individual colonies were sequenced to determine spacer integration site, orientation and length. Both the Cas1-Cas2 integrase and the Cas9-integrase supercomplex catalyzed full-site spacer integration without preferential spacer orientation (Supplementary Figure S3) and the Cas1-Cas2 integrase alone showed a higher propensity toward leader proximal integration (Supplementary Figure S4). Notably, the supercomplex showed a unique ability to generate integration products that were ∼30 bp in length regardless of the length of the integration substrates (Figure 2B–G and Supplementary Figure S5). No such conserved spacer length integration was observed when the Cas1-Cas2 integrase alone was used in these reactions; instead, Cas1-Cas2 integrase acquired spacers consistent with unprocessed substrates. These results demonstrate that the supercomplex has an intrinsic ability to process longer prespacers to a length of ∼30 bp, the correct physiological spacer length for the type II-A *S. pyogenes* system. Notably, the processing reaction by the supercomplex did not depend on sequence complementarity between the guide RNA (provided here as a single-guide RNA (sgRNA)) and the spacer sequence.

### HNH-domain of Cas9 catalyzes prespacer trimming

**Figure 3.**
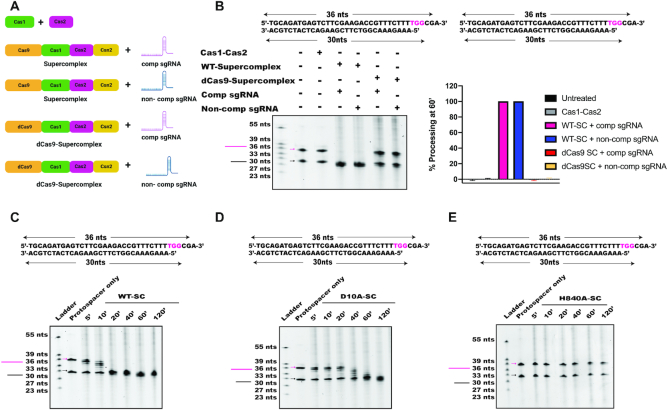
The HNH-domain of Cas9 catalyzes prespacer processing. (**A**) Schematic showing a list of complexes used to test prespacer processing by Urea-PAGE analysis. (**B**) Prespacer processing to a canonical size occurred only when prespacers were incubated with WT-supercomplex complexed with either a complementary or a noncomplementary sgRNA. Cas1-Cas2 integrase or supercomplex containing dCas9 did not show any prespacer processing. Quantification of the reactions shown have been represented as bar graphs. Error bars are representative of ±SD (*n* = 3). Time-point is 60 min. (**C–E**) Urea-PAGE gels showing kinetics of prespacer processing by supercomplex containing wild-type (WT)-Cas9 (C), RuvC-inactivated Cas9 (D10A-Cas9) (D) or HNH-inactivated Cas9 (H840A-Cas9) (E).

Since Cas1 and Cas2 alone did not exhibit DNA trimming activity in our assays, and since Csn2 has no known enzymatic function (24–26), we tested the possibility that within the supercomplex, Cas9 is responsible for prespacer trimming prior to integration (Figure 3A). DNA substrates of different lengths were incubated with Cas1-Cas2 integrase, wild-type supercomplex or supercomplex containing dCas9, a catalytically inactive version of Cas9 (Figure 3B and Supplementary Figure S6). Products of these reactions showed that no processing occurred in the presence of Cas1-Cas2 integrase alone, consistent with the results from the full-site integration assay and previous observations (Figure 3B) (4,15). In the presence of the supercomplex, however, prespacers were processed to ∼30 bp products in the presence of either a complementary (targeting) or noncomplementary (nontargeting) sgRNA. No processing was observed when a supercomplex containing catalytically deactivated Cas9 (dCas9) was used, suggesting that Cas9 nuclease activity is directly responsible for prespacer processing (Figure 3B).

*Streptococcus pyogenes* Cas9 contains two endonuclease domains (RuvC and HNH) that together generate DNA double-strand breaks by cleaving opposite strands of the DNA helix (3,27,28). To determine which of these nuclease active sites is required for prespacer processing, we tested mutant versions of the supercomplex containing Cas9 with either a RuvC-inactivating mutation (D10A) or an HNH-inactivating mutation (H840A) (2,3). Results showed that prespacers were processed by supercomplex containing a wild-type Cas9 or the RuvC-inactivating mutation in Cas9, but processing was abolished by the HNH-inactivating mutation in Cas9 (Figure 3C–E). These results indicate that within the supercomplex, Cas9′s HNH domain, defined originally as an endonuclease (2,3), catalyzes exonucleolytic prespacer trimming to the physiologically relevant CRISPR spacer length.

### Comparing the role of Cas9 for spacer integration *in vitro* and *in vivo*

**Figure 4.**
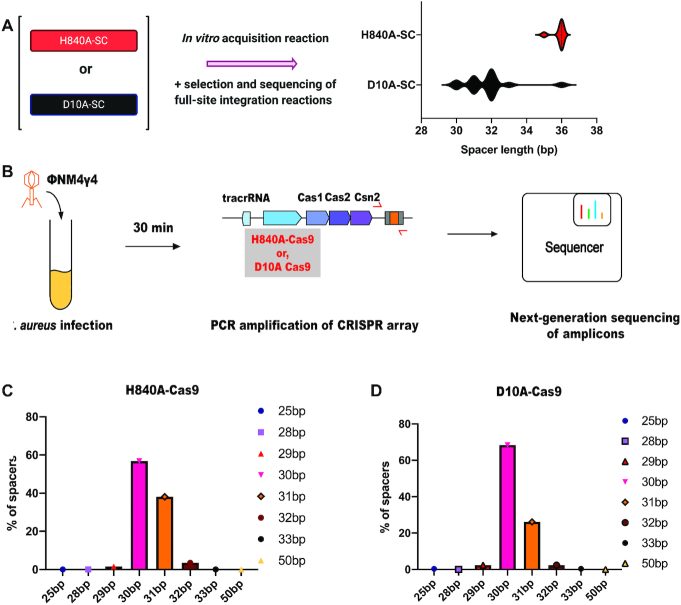
Cas9 is required for prespacer processing *in vitro* but not in cells. (**A**) Length of integrated spacers upon full-site integration reactions *in vitro* by supercomplex containing H840A-Cas9 or D10A-Cas9 (*n* = 7 for H840A-SC; *n* = 15 for D10A-SC). Integration events using supercomplex with H840A-Cas9 led to more ectopic and recombination events, thereby lowering the number of faithful integration events. (**B**) Schematic of the experimental system used for studying spacer length acquired upon phage infection by D10A-Cas9 and H840A-Cas9. (**C**) Deep-sequencing analysis of spacer lengths acquired 30 min after phage infection of *S. aureus* RN4220 harboring either H840A-Cas9 or (**D**) D10A-Cas9.

We next tested whether prespacer processing by the supercomplex affects spacer integration *in vitro*. Purified samples of supercomplex containing RuvC-inactivated Cas9 or HNH-inactivated Cas9 were tested using the plasmid transformation screen described above (Figure 2A). Both of these complexes generated full-site integration events, but only the RuvC-inactivated supercomplex produced ∼30 bp insertions (Figure 4A), consistent with the results from substrate trimming (Figure 3D,E). The HNH-inactivated supercomplex generated unprocessed spacers, and more insertions occurred at ectopic sites. To test whether Cas9 is responsible for prespacer trimming *in vivo*, we performed ΦNM4γ4 phage (9) challenge experiments with *S. aureus* RN4220 cultures harboring a plasmid containing the *S. pyogenes* type II-A CRISPR-Cas system with either a D10A (RuvC-inactivating) or a H840A (HNH-inactivating) Cas9 variant (Figure 4B). With both of these variants, we analyzed the sizes of phage-derived spacers acquired into the CRISPR locus using next-generation sequencing. We were surprised to find that both these variants of Cas9 resulted in integration of 30–31 bp spacers as detected in this assay (Figure 4C,D). Since the deep-sequencing assay is not quantitative, it is possible that fewer acquisition events occurred with the H840A Cas9 mutant strains and that the observed integration events resulted from 30 bp spacers generated by other cellular nucleases. It therefore seems possible that Cas9 is not the only nuclease that can catalyze prespacer trimming *in vivo*. These results are consistent with previous reports showing the dispensability of Cas9′s nuclease activity for adaptation (9,10) and highlight Cas9′s contribution to an early step in the integration pathway.

### Role of crRNA spacer sequence for substrate trimming by the supercomplex

**Figure 5.**
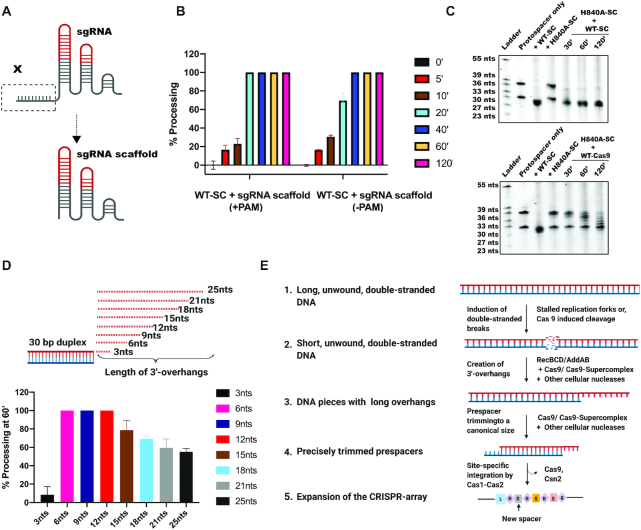
Prespacer processing by supercomplex is independent of crRNA spacer sequence. (**A**) Schematic showing the design of sgRNA scaffold. (**B**) WT-supercomplex, in complex with sgRNA-scaffold, trims PAM-containing prespacers with relatively faster kinetics. Bar graphs represent the percentage processing with time, for prespacers either containing or lacking a PAM. Error bars represent ± SD; *n* = 3. (**C**) Rescue of *in vitro* prespacer processing by supplementing H840A-supercomplex with either WT-supercomplex or WT-Cas9. (**D**) Design of substrates with different lengths of 3′-overhangs for assessing substrate preference by supercomplex (top). Quantification of processing by the WT-supercomplex for prespacers containing different 3′-overhang lengths are represented as bar graphs (bottom); *n* = 3 and error bars represent ± SD. (**E**) Model of prespacer processing by the Cas9-integration supercomplex. Self-derived spacers are most frequently derived from genomic loci with a high frequency of generating double-stranded breaks. In *S. pyogenes*, such double-stranded breaks are repaired by AddAB, which creates DNA pieces with long 3′-overhangs. These products can be captured by the Cas9-supercomplex along with additional cellular nucleases and processed to a canonical size for integration by the Cas1-Cas2 integrase for site-specific integration.

During type II-A CRISPR target interference, the Cas9:tracrRNA:crRNA complex binds to target DNA at a site complementary to the 20-nucleotide guide sequence of the crRNA and adjacent to a PAM sequence (NGG for *S. pyogenes* Cas9) (27,29). We tested whether a similar mechanism of DNA recognition influences binding of DNA integration substrates by first evaluating the effect of deleting the crRNA guide sequence on prespacer processing (Figure 5A). Under these conditions, the RNA scaffold lacking the guide sequence maintains its ability to assemble with Cas9 (27). Surprisingly, the supercomplex bearing a truncated sgRNA without the 20-nucleotide spacer sequence maintained prespacer processing activity (Figure 5B and Supplementary Figure S7). In the absence of a PAM, prespacer processing was ∼2-fold slower relative to a PAM-containing prespacer (Supplementary Figures S7 and S8). Together, these data show that DNA binding by the supercomplex is independent of Cas9′s RNA-guided DNA targeting mechanism, enabling integration substrate precursors with a wide range of sequences to be processed for acquisition into the CRISPR array. We also performed experiments to test whether *in vitro* prespacer trimming in reactions containing H840A-Cas9-containing supercomplex could be restored by either wild-type Cas9 or wild-type supercomplex, both containing an intact sgRNA scaffold. Prespacer trimming could be rescued in both cases but with comparatively faster trimming kinetics in case of the supercomplex (Figure 5C). In order to further understand whether the trimming activity by Cas9 requires the association of Cas1-Cas2-Csn2, we performed these assays with supercomplex containing a sgRNA scaffold and compared it with Cas9 complexed with sgRNA scaffold (Supplementary Figures S7 and S9). The results showed that Cas9 is capable of prespacer trimming even in the absence of the adaptation module (12), but this activity is enhanced within the supercomplex (Supplementary Figure S9).

Genomic loci with high propensity toward stalled replication forks or double-stranded breaks are sources of spacers generated in bacteria (30). Double-stranded DNA breaks are repaired by enzymes including RecBCD or AddAB that processively trim the DN5A ends, generating short fragments with 3′-overhangs that could serve as substrates for CRISPR integration (1,4). To test the substrate preference for the supercomplex, we designed a series of prespacer substrates containing 3′-overhangs ranging from 3 to 25 nucleotides (Figure 5D). After incubation with the Cas9-integrase supercomplex, we found that prespacers containing 3′-overhangs between 6–12 nucleotides in length were completely processed to ∼30 bp products. However, prespacers with longer 3′-overhangs were processed with decreasing efficiency (Figure 5D). Additionally, the supercomplex had minimal ability to process long double-stranded prespacer substrates (Supplementary Figure S10). These findings suggest that *S. pyogenes* Cas9 can convert DNA fragments with short 3′-overhangs into properly trimmed substrates for integration into the CRISPR array (Figure 5E).

## DISCUSSION

Our results suggest that in the type II-A CRISPR-Cas system of *S. pyogenes*, the Cas9-integrase supercomplex binds to prespacers, trims them to a size of ∼30 bp and catalyzes integration into the CRISPR array. Cas9 is a precise RNA-guided endonuclease for generating double-stranded DNA-breaks during CRISPR-based adaptive immunity (2,27,28). The present work uncovers a distinct new catalytic behavior of Cas9 with potential significance in the adaptation pathway. We show that the HNH domain of Cas9 functions as a guide RNA-independent exonuclease in the context of prespacer trimming and helps in generating correctly sized substrates for integration into the CRISPR array by the Cas1-Cas2 integrase. The prespacer trimming activity of Cas9 is enhanced when in complex with Cas1-Cas2-Csn2 and is not reliant on guide RNA-based DNA binding. This guide RNA-independent mode of substrate binding has biological significance, because it would enable Cas9 to generate processed integration substrates from previously unencountered DNA sequences.

Complex formation between Cas1, Cas2, Csn2 and Cas9 from the type II-A system has been reported previously (9,13,31), but the factors determining the stability of this complex *in vitro* need to be understood and explored further. Csn2 has been reported to act as an interface for the interaction of Cas9 with Cas1-Cas2 for supercomplex assembly (13,31). Additionally, Cas9 has been shown to associate with Cas1-Cas2-Csn2 by a DNA tether (13), indicating that both protein–protein interactions as well as DNA–protein interactions contribute to the stability of the supercomplex *in vitro*. Our experiments indicate that while the supercomplex can form in the absence of a prespacer DNA, it is most stable when both sgRNA and a prespacer DNA are present. Since prespacer trimming is mediated by both Cas9 alone as well as an intact supercomplex, it can be argued that prespacer trimming and adaptation can be mediated by a sequential interaction of Cas9 and the adaptation module respectively. However, the trimming activity of Cas9 within the supercomplex is enhanced, indicating that association of Cas9 with Cas1-Cas2-Csn2 accelerates the kinetics and/or extent of prespacer processing.

While the mechanism of prespacer generation in type II-A CRISPR-Cas systems is not well understood, the machinery responsible for prespacer trimming has been reported for a few other CRISPR-Cas systems. In the type I-E system from *E. coli*, DnaQ exonucleases have been shown to perform prespacer processing, with the canonical sized spacers being integrated by Cas1-Cas2 at the leader-repeat junction in the correct orientation (32). In the type I-A and type I-C systems, Cas4 nuclease is required for PAM-recognition, prespacer processing and directional spacer integration (33,34). In this study, we have shown that in the type II-A system of *S. pyogenes*, Cas9 is involved in prespacer trimming but the precise mechanisms underlying PAM specificity of prespacer processing and directional integration of spacer into the CRISPR array remain to be explored. Sequencing analysis from full-site integration experiments showed that spacers integrated by the supercomplex are always trimmed to 30 bp but the integration is neither specific to the leader-repeat junction nor are the spacers always integrated in the physiologically relevant orientation. The supercomplex might restrict site-specific interaction of Cas1-Cas2 with the leader-repeat junction and points toward a kinetic timeline where the dissociation of Cas9 and Csn2 from the supercomplex would make the Cas1-Cas2 integrase available for site-specific integration. The dissociation of Cas9 from the supercomplex would leave behind a Cas1-Cas2-Csn2 complex, the structure of which has recently been reported (13). In the Cas1-Cas2-Csn2 structure, Csn2 wraps around the bound spacer, thereby restricting its access to both Cas1 and Cas2. Csn2 must eventually slide off the bound spacer and hand the processed substrate to Cas1-Cas2 for site-specific integration (15). This sequence of events is similar to what occurs with Cas4 in type I systems, where its association with Cas1-Cas2 results in the formation of a higher order structure for prespacer trimming and is followed by its dissociation from the Cas1-Cas2 complex prior to spacer integration (35).

Since the nuclease activity of Cas9 is dispensable for acquiring canonical-sized spacers upon phage-infection, we believe that other cellular nucleases in addition to Cas9 can mediate prespacer processing. Cas9′s capacity for prespacer trimming may enable horizontal gene transfer of CRISPR-Cas systems into hosts with diverse genetic backgrounds (36) by avoiding absolute reliance on specific host nucleases for immunity acquisition. Taken together, this study establishes a possible role for the Cas9-integrase supercomplex that is upstream of the final integration event and outlines a novel nuclease activity of Cas9 in creating immunological memory in the type II-A CRISPR-Cas systems.

## DATA AVAILABILITY

The raw deep sequencing data collected for this study is available under the bioproject ID PRJNA670708.

## Supplementary Material

gkab123_Supplemental_File
