# Supplementary material for: A CRISPR-Cas9–integrase complex generates precise DNA fragments for genome integration
Source: Nucleic Acids Res. 2021 Mar 8;49(6):3546–56. doi: 10.1093/nar/gkab123 (PMC8034620; doi:10.1093/nar/gkab123)
Supplement: gkab123_Supplemental_File [file gkab123_supplemental_file.pdf]

A CRISPR-Cas9-integrase complex generates precise DNA fragments for genome integration

Authors: Shrutee Jakhanwal[1,2,4,6], Brady F. Cress[1,2,4,6], Pascal Maguin[9], Marco J. Lobba[1,2,3.6], Luciano A. Marraffini[9], Jennifer A. Doudna[1-8*]

Supplementary data

Supplementary Figure 1. Purification and characterization of the Cas9-integration supercomplex.
Supplementary Figure 2. Increasing spacer length does not affect CRISPR interference against phage.
Supplementary Figure 3. Orientation of spacers upon full-site integration by either Cas1-Cas2 integrase or the supercomplex.
Supplementary Figure 4. Cas1-Cas2 integrase mediates leader proximal integration *in vitro*.
Supplementary Figure 5. Trimming pattern of prespacers by the supercomplex.
Supplementary Figure 6. Cas1-Cas2 integrase or dCas9-supercomplex cannot trim prespacers.
Supplementary Figure 7. Prespacer processing by supercomplex is independent of spacer sequence.
Supplementary Figure 8. Urea-PAGE analysis of prespacer processing for substrates either containing or lacking a PAM.
Supplementary Figure 9. Substrate cleavage by wild-type, D10A and H840A Cas9 complexed with an sgRNA-scaffold.
Supplementary Figure 10. Determination of substrate preference by the supercomplex.
Supplementary Table 1. Oligonucleotide sequences used for PCR and molecular cloning in the *S. aureus (in vivo)* experiments.
Supplementary Table 2. Prespacers used for the full-site integration selection assay.
Supplementary Table 3. Number of colonies analyzed by Sanger sequencing upon full-site integration of different prespacers by Cas1-Cas2 integrase and the supercomplex.
Supplementary Table 4. Prespacers used for *in vitro* processing experiments
Supplementary Table 5. Prespacers used for experiments shown in Figure 5d and Supplementary Figure 10a.
Supplementary Table 6. Prespacers used for experiments shown in Supplementary Figure 10b.

Supplementary Figure 1.

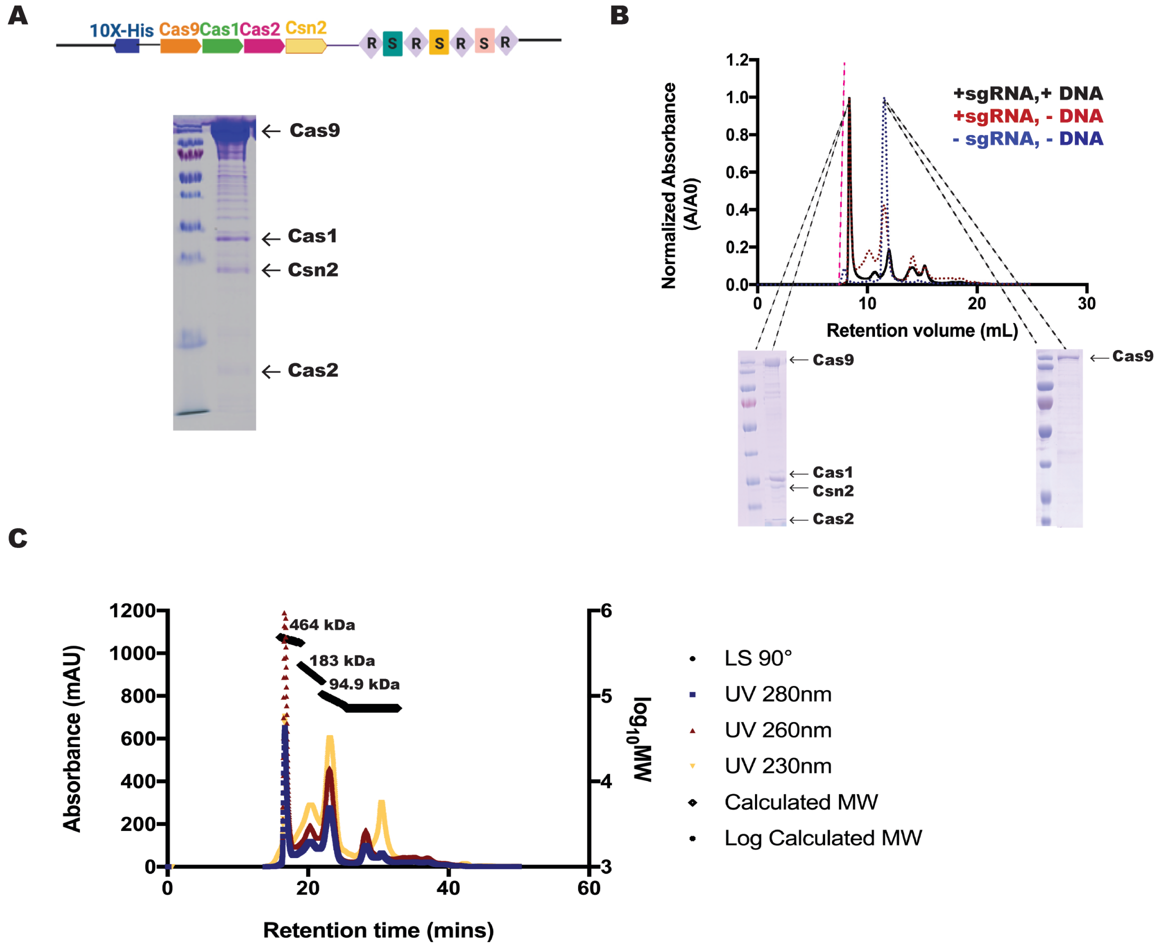

Supplementary Figure 1: Purification and characterization of the Cas9-integration supercomplex. **(A)** SDS-PAGE gel showing purification of the supercomplex from a Heparin column. **(B)** Stability of the supercomplex depends on the presence of sgRNA and a spacer. Purification profile of the supercomplex in the absence of an sgRNA and a pre-spacer DNA (*blue*), in the presence of only an sgRNA (*red*) and both sgRNA and a pre-spacer DNA (*black*). SDS-PAGE gels for the major peaks are shown below. Dotted line marks the void volume of the column. **(C)** Molecular weight of the supercomplex was determined by size-exclusion chromatography coupled to dual angle laser light scattering (SEC-DALLS). The observed molecular weight of the supercomplex containing Cas1-Cas2-Csn2 and Cas9 was 464kDa. The other peaks correspond to sub-constituents of the supercomplex.

Supplementary Figure 2.

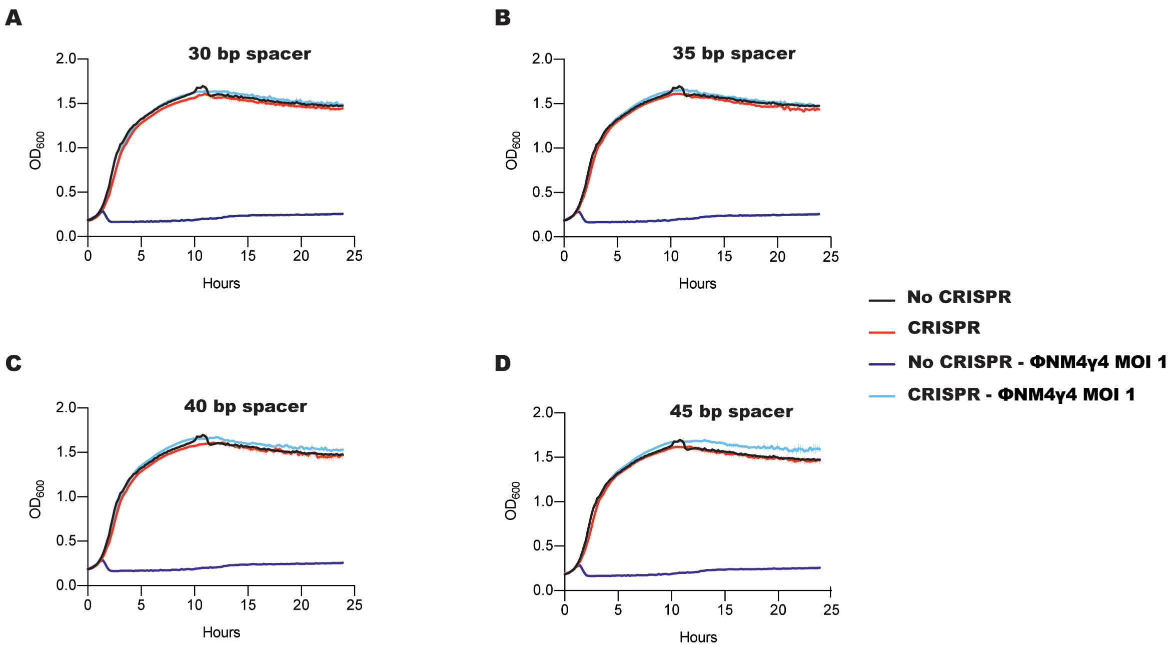

Supplementary Figure 2: Increasing spacer length does not affect CRISPR interference against phage. Growth curves of *S. aureus* RN4220 infected with ΦNM4γ4 harboring an empty plasmid or a plasmid containing the *S. pyogenes* type II-A CRISPR-Cas system (ΔCas1, ΔCas2, and ΔCsn2) with spacer-lengths of **(A)** 30bp **(B)** 35bp **(C)** 40bp and **(D)** 45bp. Growth was monitored by recording the OD_{600} of the cultures over time. The data for empty plasmid (*black*) and for empty plasmid infected with phage (*blue*) are the same in all four graphs. Mean ±SD of three replicates is reported.

Supplementary Figure 3.

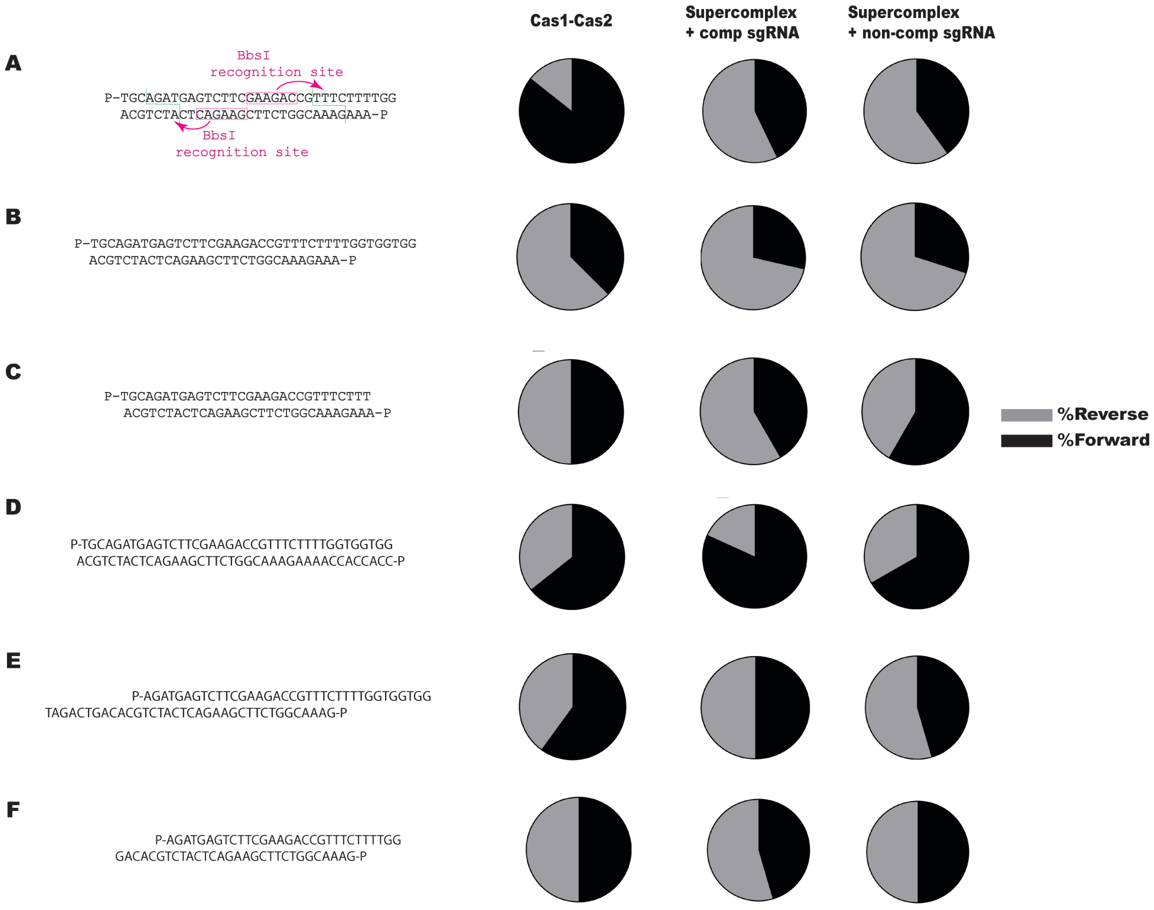

Supplementary Figure 3: Orientation of spacers upon full-site integration by either Cas1-Cas2 integrase or the supercomplex. Sanger sequencing of the integrated spacers showed no orientation preference *in vitro* either by Cas1-Cas2 integrase or the supercomplex. Results **(A-F)** have been shown for the six different substrates discussed in Figure 2. Forward orientation indicates 5'-end of substrates oriented toward leader, while reverse orientation indicates the opposite. Black represents forward orientation and grey represents reverse orientation. BbsI recognition site and cleavage pattern has been marked for the first prespacer substrate. The pink boxes highlight the BbsI recognition sites, while the green lines indicate the cleavage pattern. The arrows indicate directional cleavage by BbsI outside of the recognition site.

Supplementary Figure 4.

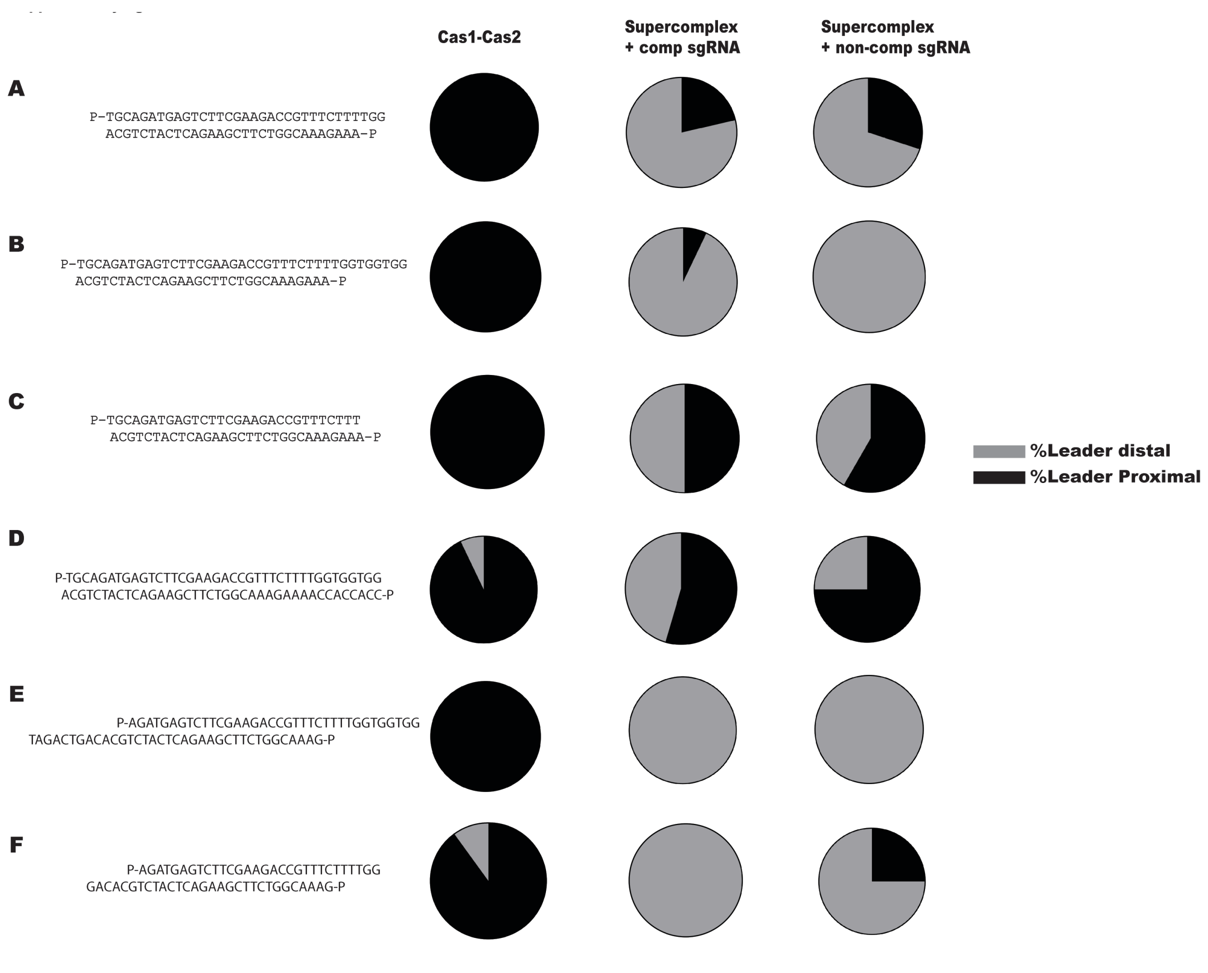

Supplementary Figure 4: Cas1-Cas2 integrase mediates leader proximal integration *in vitro*. Cas1-Cas2 integrase but not supercomplex displays a propensity toward leader-proximal integrations *in vitro*. Percentage integrations at leader proximal repeat *(black)* or leader distal repeat (also known as ectopic integrations) *(grey)* have been represented in panels A-F for the prespacer substrates shown in Figure 2. Integrations occurring at the first repeat within the CRISPR array have been referred to as integrations at the 'leader proximal repeat' and integrations occurring at any other repeat within the CRISPR array have been referred to as integrations at the 'leader distal repeat'.

Supplementary Figure 5.

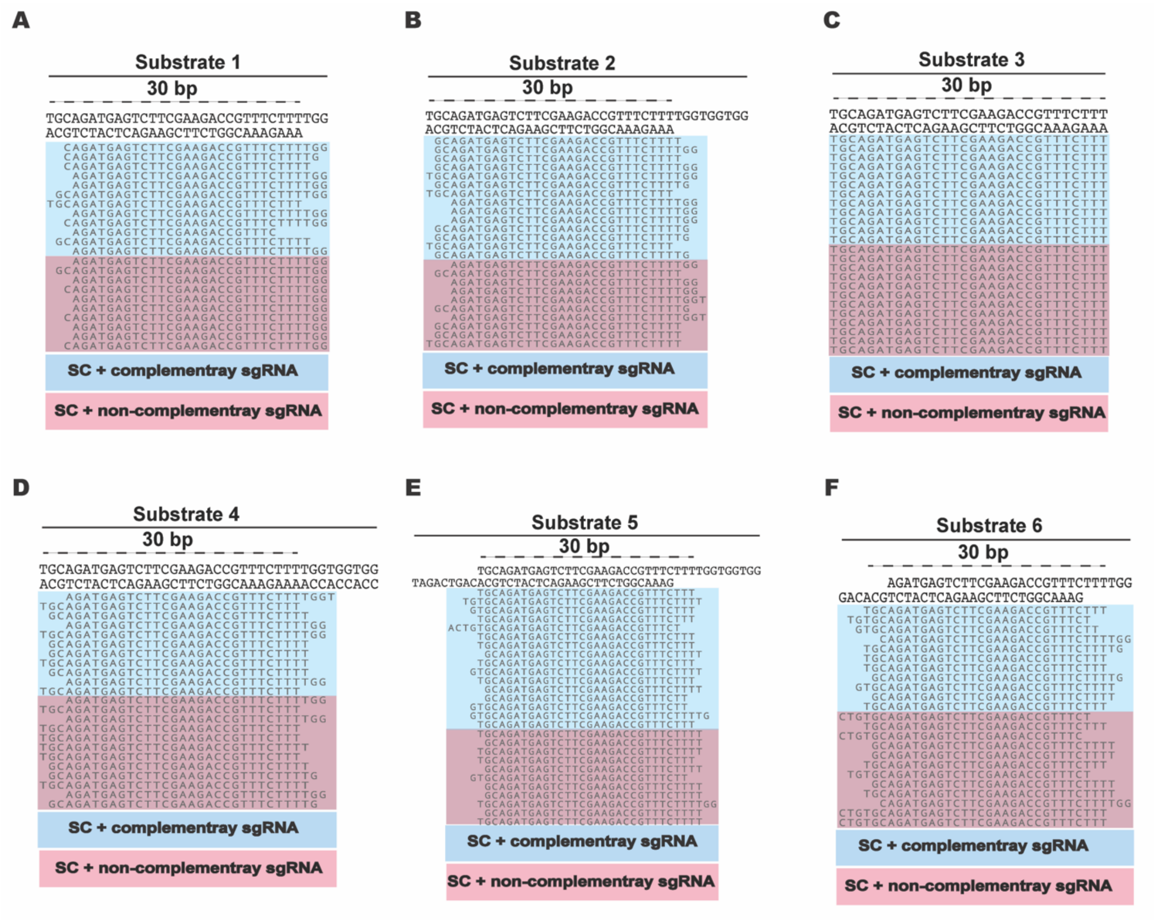

Supplementary Figure 5: Trimming pattern of prespacers by the supercomplex. Supercomplex trims nucleotides from either end of the prespacer substrates to a canonical size of ~30bp, irrespective of sequence complementarity between crRNA and the prespacer; SC: Supercomplex.

Supplementary Figure 6.

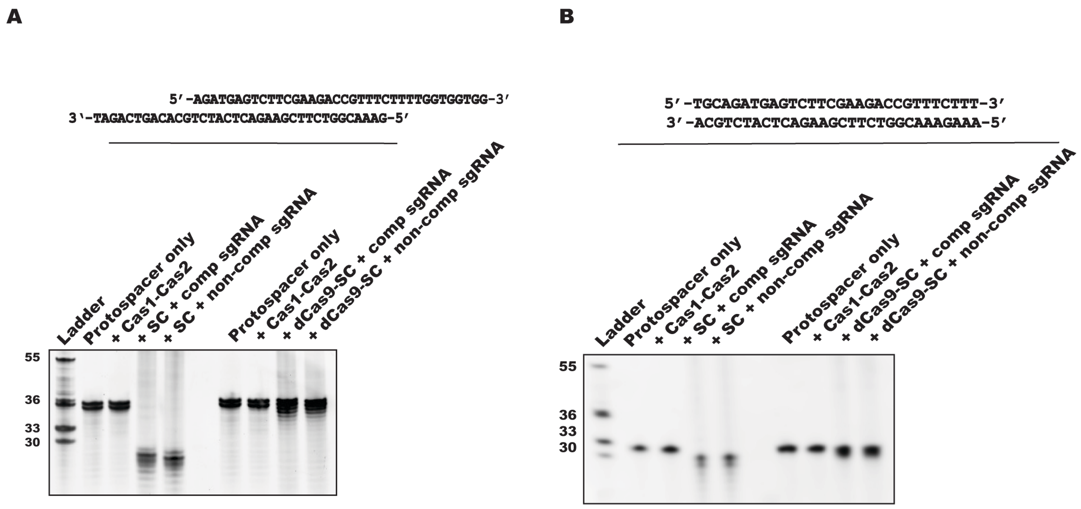

Supplementary Figure 6: Cas1-Cas2 integrase or dCas9-supercomplex cannot trim prespacers. **(A)** Gels showing prespacer size upon incubation with Cas1-Cas2 integrase, WT-supercomplex or a supercomplex with dCas9. Results are shown for a prespacer with 3’-overhangs and **(B)** a 30bp-double stranded prespacer.

Supplementary Figure 7.

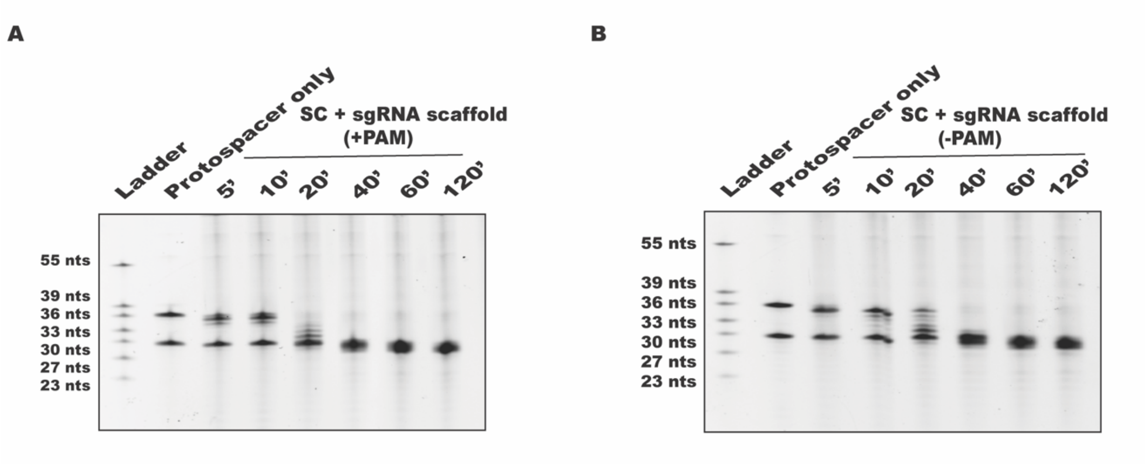

Supplementary Figure 7: Prespacer processing by supercomplex complexed with sgRNA scaffold. (A) Gel showing kinetics of processing by supercomplex containing sgRNA scaffold for a substrate containing a PAM or **(B)** lacking a PAM.

Supplementary Figure 8.

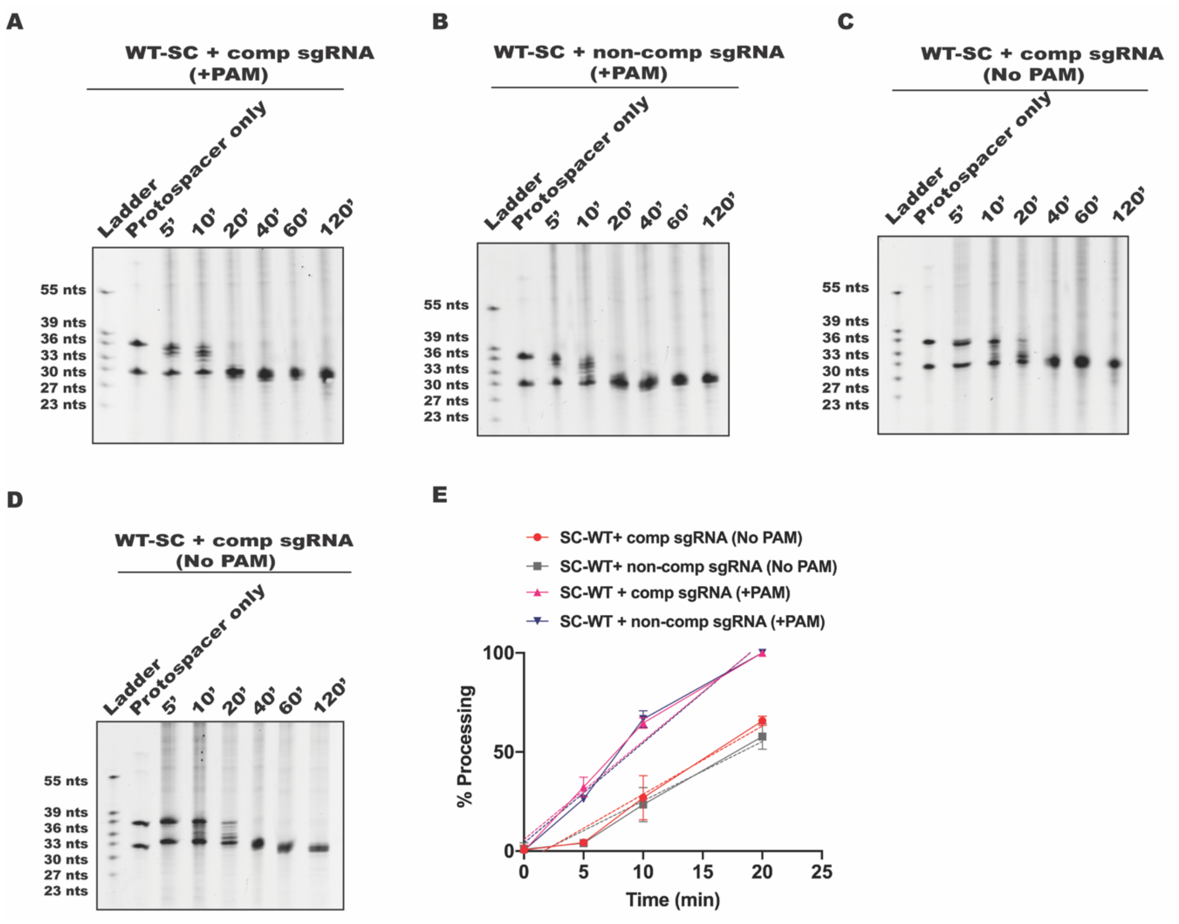

Supplementary Figure 8: Urea-PAGE analysis of prespacer processing for substrates either containing or lacking a PAM. (A) Gel showing time-points for processing of a PAM-containing prespacer by WT-supercomplex containing a complementary sgRNA or a **(B)** non-complementary sgRNA. **(C)** Gel showing processing kinetics for a PAM-less prespacer by WT-supercomplex in association with a complementary or **(D)** a non-complementary sgRNA. The prespacers used for these experiments were unlabeled and gels were visualized by SYBR-gold staining. **(E)** Kinetics of processing for prespacers containing or lacking a PAM by a WT-supercomplex. Percentage of processed prespacers is plotted as a function of time. The rates of processing obtained from fitting the curves using simple linear regression are 4.960 ± 0.2788 min^{-1} for WT-SC + comp sgRNA (+PAM), 5.060 ± 0.3090 min^{-1} for WT-SC + non-comp sgRNA (+PAM), 3.438 ± 0.2958 min^{-1} for WT-SC + comp sgRNA (-PAM) and 2.996 ± 0.2741 min^{-1} for WT-SC + non-comp sgRNA (-PAM) *n-=3*. Dotted lines represent the fit.

Supplementary Figure 9.

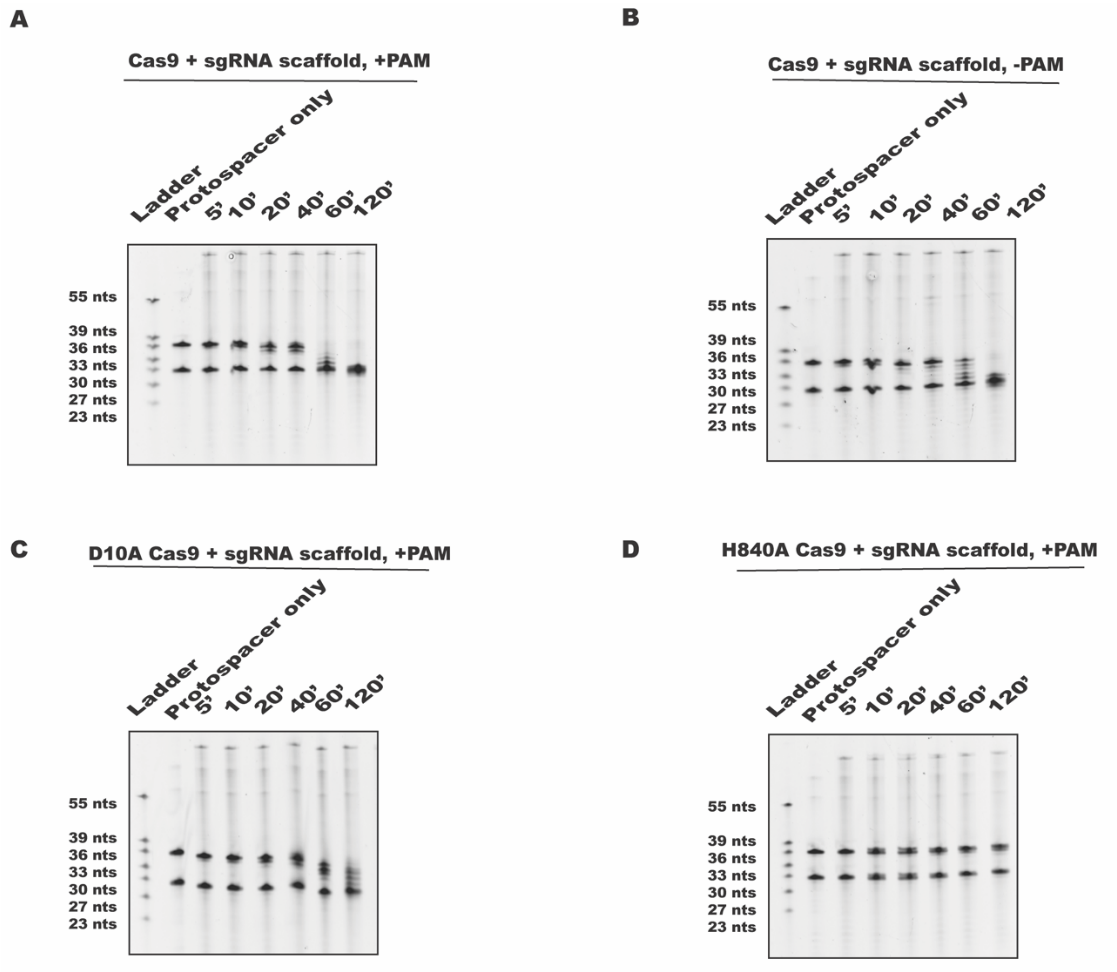

Supplementary Figure 9: Substrate cleavage by wild-type, D10A and H840A Cas9 complexed with an sgRNA-scaffold. (A) Gel showing kinetics of cleavage by WT-Cas9 complexed with sgRNA scaffold for a substrate containing a PAM or **(B)** lacking a PAM. The prespacers used for these experiments were unlabeled and gels were visualized by SYBR-gold staining **(C)** Kinetics of cleavage for a PAM-containing substrate by a dRuvC-Cas9 (D10A) and **(D)** a dHNH-Cas9 (H840A) complexed with sgRNA scaffold.

Supplementary Figure 10.

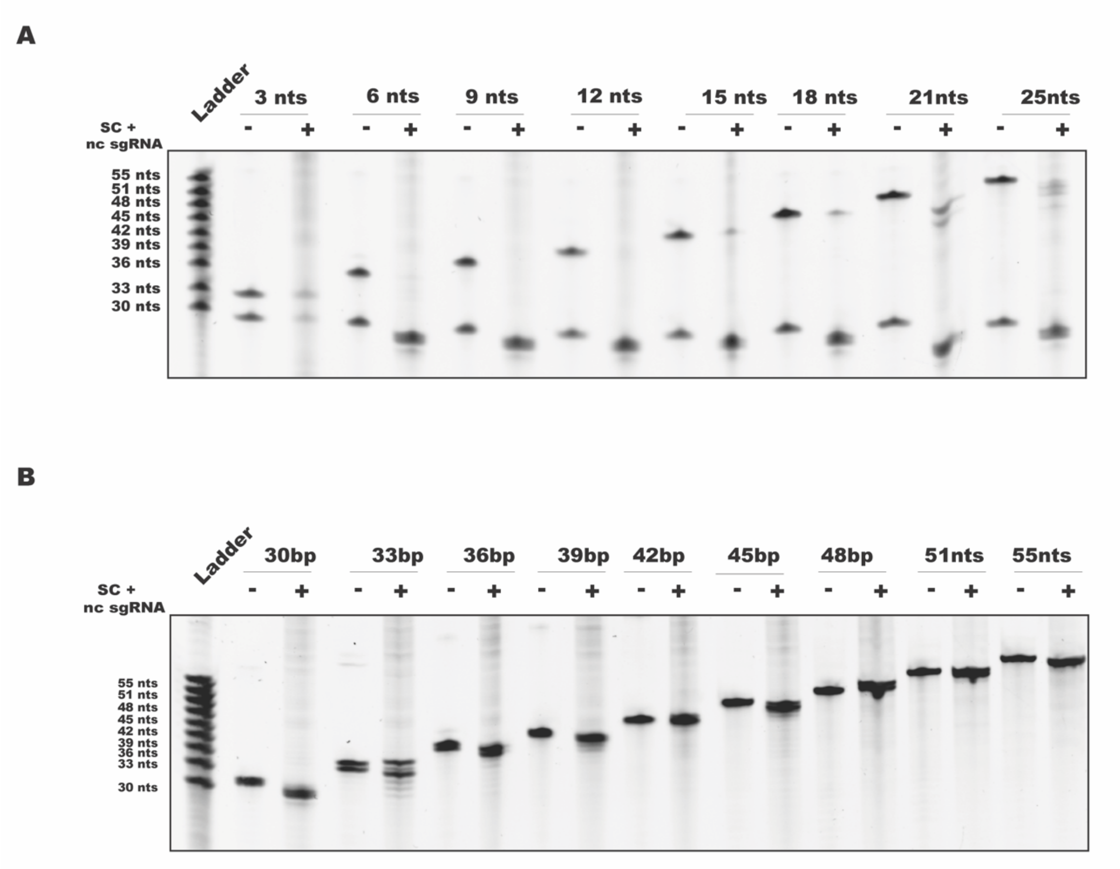

Supplementary Figure 10: Determination of substrate preference by the supercomplex. (A) Urea-PAGE gel showing processing of substrates containing different lengths of 3'-overhangs and **(B)** for double-stranded substrates of different lengths. All the prespacers were unlabeled and gels were visualized by SYBR-gold staining.

Supplementary Table 1: Oligonucleotide sequences used for PCR and molecular cloning in the *S. aureus (in vivo)* experiments.

Oligo	Sequence
B295	5'-TCAGTCACCTCCTAGCTGACTCAA
B337	5'-GACGCTATTTGTGCCGATAGCTAAGCCTATTGAGTATTTC
B338	5'-GAAATACTCAATAGGCTTAGCTATCGGCACAAATAGCGTC
B339	5'-GGAAACTTTGTGGAACAATGGCATCGACATCATAATCACT
B340	5'-AGTGATTATGATGTCGATGCCATTGTTCCACAAAGTTTCC
H061	5'-TTTTAGGAGGCAAAAATGGATAAGAAATACTCAATAGGCT
H064	5'-GTATTTCTTATCCATTTTTGCCTCCTAAAATAAAAAGTTT
H295	5'-AACACGCATTGATTTGAGTCAGC
H296	5'-TCCTAGCTGACTCAAATCAATGCG
PM168	5'-NNNNNAGTGCGATTACAAAATTTTTTAGAC
PM375	5'-NNNNNAAAACAGCATAGCTCTAAAACG
PM376	5'-NNNNNAAAACAGCATAGCTCTAAAACA
PM377	5'-NNNNNAAAACAGCATAGCTCTAAAACT
PM1206	5'-AAACAGAAAAGTAAGAGTAATCGGAGACGATTTCG
PM1207	5'-AAAACGAAATCGTCTCCGATTACTCTTACTTTTCT
PM1266	5'-AAACATGCGAGAAAAGTAAGAGTAATCGGAGACGATTTCG
PM1267	5'-AAAACGAAATCGTCTCCGATTACTCTTACTTTTCTCGCAT
PM1268	5'-AAACACACAATGCGAGAAAAGTAAGAGTAATCGGAGACGATTTCG
PM1269	5'-AAAACGAAATCGTCTCCGATTACTCTTACTTTTCTCGCATTGTGT
PM1270	5'- AAACATGAAACACAATGCGAGAAAAGTAAGAGTAATCGGAGACGAT TTCG
PM1271	5'- AAAACGAAATCGTCTCCGATTACTCTTACTTTTCTCGCATTGTGTTTC AT

Supplementary Table 2: Prespacers used for the full-site integration selection assay (Figure 2B-G, Supplementary Figures 3-5)

Description	Sequence
Substrate1_T	5'-P/TGCAGATGAGTCTTCGAAGACCGTTTCTTTTGG-3'
Substrate1_B	5'-P/AAAGAAACGGTCTTCGAAGACTCATCTGCA-3'
Substrate2_T	5'-P/TGCAGATGAGTCTTCGAAGACCGTTTCTTTTGGTGGTGG-3'
Substrate2_B	5'-P/AAAGAAACGGTCTTCGAAGACTCATCTGCA-3'
Substrate3_T	5'-P/ TGCAGATGAGTCTTCGAAGACCGTTTCTTT-3'
Substrate3_B	5'-P/AAAGAAACGGTCTTCGAAGACTCATCTGCA-3'
Substrate4_T	5'-P/TGCAGATGAGTCTTCGAAGACCGTTTCTTTTGGTGGTGG-3'
Substrate4_B	5'-P/CCACCACCAAAAGAAACGGTCTTCGAAGACTCATCTGCA-3'
Substrate5_T	5'-P/ AGATGAGTCTTCGAAGACCGTTTCTTTTGGTGGTGG-3'
Substrate5_B	5'-P/ GAAACGGTCTTCGAAGACTCATCTGCACAGTCAGAT-3'
Substrate6_T	5'-P/ AGATGAGTCTTCGAAGACCGTTTCTTTTGG-3'
Substrate6_B	5'-P/ GAAACGGTCTTCGAAGACTCATCTGCACAG-3'

Supplementary Table 3. Number of colonies analyzed by Sanger sequencing upon full-site integration of different prespacers by Cas1-Cas2 integrase and the supercomplex (Figure 2, Supplementary Figures 3-5).

Substrate	Complex	Number of colonies
Substrate1	Cas1-Cas2 integrase<br>Supercomplex + comp sgRNA<br>Supercomplex + non-comp sgRNA	7<br>14<br>10
Substrate2	Cas1-Cas2 integrase<br>Supercomplex + comp sgRNA<br>Supercomplex + non-comp sgRNA	8<br>14<br>10
Substrate3	Cas1-Cas2 integrase<br>Supercomplex + comp sgRNA<br>Supercomplex + non-comp sgRNA	12<br>12<br>12
Substrate4	Cas1-Cas2 integrase<br>Supercomplex + comp sgRNA<br>Supercomplex + non-comp sgRNA	14<br>11<br>12
Substrate5	Cas1-Cas2 integrase<br>Supercomplex + comp sgRNA<br>Supercomplex + non-comp sgRNA	15<br>16<br>11
Substrate6	Cas1-Cas2 integrase<br>Supercomplex + comp sgRNA<br>Supercomplex + non-comp sgRNA	10<br>11<br>12

Supplementary Table 4: Prespacers used for *in vitro* processing experiments (Figure 3B-E; Figure 4A; Figure 5B, C and Supplementary Figures 7-9 *unless stated otherwise*)

Description	Sequence
36nts-Top strand (+PAM)	5'-P/TGCAGATGAGTCTTCGAAGACCGTTTCTTTTGGCGA-3'
36nts-Top strand (-PAM)	5'-P/TGCAGATGAGTCTTCGAAGACCGTTTCTTTCAGCGA-3'
30nts-Bottom strand	5'-P/AAAGAAACGGTCTTCGAAGACTCATCTGCA-3'
36nts-Top strand	5'-P/AGATGAGTCTTCGAAGACCGTTTCTTTTGGTGGTGG -3' (Supplementary Figure 6A)
36nts-Bottom strand	5'-P/GAAACGGTCTTCGAAGACTCATCTGCACAGTCAGAT-3' (Supplementary Figure 6A)
30nts-Top strand	5'-P/TGCAGATGAGTCTTCGAAGACCGTTTCTTT-3' (Supplementary Figure 6B)
30nts-Bottom strand	5'-P/AAAGAAACGGTCTTCGAAGACTCATCTGCA-3' (Supplementary Figure 6B)

Supplementary Table 5: Prespacers used for experiments shown in Figure 5D and Supplementary Figure 10A.

Description	Sequence
30nts _B	5'-P/AAAGAAACGGTCTTCGAAGACTCATCTGCA-3'
33nts _T	5'-P/TGCAGATGAGTCTTCGAAGACCGTTTCTTTTGG-3'
36nts _T	5'-P/TGCAGATGAGTCTTCGAAGACCGTTTCTTTTGGCGA-3'
39nts_T	5'-P/TGCAGATGAGTCTTCGAAGACCGTTTCTTTTGGCGAAGA-3'
42nts_T	5'-P/TGCAGATGAGTCTTCGAAGACCGTTTCTTTTGGCGAAGACTC-3'
45nts_T	5'-P/TGCAGATGAGTCTTCGAAGACCGTTTCTTTTGGCGAAGACTCATC-3'
48nts_T	5'-P/TGCAGATGAGTCTTCGAAGACCGTTTCTTTTGGCGAAGACTCATCTGC-3'
51nts_T	5'-P/TGCAGATGAGTCTTCGAAGACCGTTTCTTTTGGCGAAGACTCATCTGCACG-3'
55nts_T	5'-P/TGCAGATGAGTCTTCGAAGACCGTTTCTTTTGGCGAAGACTCATCTGCACGACTC-3'

Supplementary Table 6: Prespacers used for experiments shown in Supplementary Figure 10B.

Description	Sequence
30nts_T	5’-P/TGCAGATGAGTCTTCGAAGACCGTTTCTTT-3’
30nts_B	5’-P/AAAGAAACGGTCTTCGAAGACTCATCTGCA-3’
33nts_T	5’-P/TGCAGATGAGTCTTCGAAGACCGTTTCTTTTGG-3’
33nts_B	5’-P/CCAAAAGAAACGGTCTTCGAAGACTCATCTGCA-3’
36nts_T	5’-P/TGCAGATGAGTCTTCGAAGACCGTTTCTTTTGGCGA-3’
36nts_B	5’-P/TCGCCAAAAGAAACGGTCTTCGAAGACTCATCTGCA-3’
39nts_T	5’-P/TGCAGATGAGTCTTCGAAGACCGTTTCTTTTGGCGAAGA-3’
39nts_B	5’-P/TCTTCGCCAAAAGAAACGGTCTTCGAAGACTCATCTGCA-3’
42nts_T	5’-P/TGCAGATGAGTCTTCGAAGACCGTTTCTTTTGGCGAAGACTC-3’
42nts_B	5’-P/GAGTCTTCGCCAAAAGAAACGGTCTTCGAAGACTCATCTGCA-3’
45nts_T	5’-P/TGCAGATGAGTCTTCGAAGACCGTTTCTTTTGGCGAAGACTCATC-3’
45nts_B	5’-P/GATGAGTCTTCGCCAAAAGAAACGGTCTTCGAAGACTCATCTGCA-3’
48nts_T	5’-P/TGCAGATGAGTCTTCGAAGACCGTTTCTTTTGGCGAAGACTCATCTGC-3’
48nts _B	5’-P/GCAGATGAGTCTTCGCCAAAAGAAACGGTCTTCGAAGACTCATCTGCA-3’
51nts_T	5’-P/TGCAGATGAGTCTTCGAAGACCGTTTCTTTTGGCGAAGACTCATCTGCACG-3’
51nts _B	5’-P/CGTGCAGATGAGTCTTCGCCAAAAGAAACGGTCTTCGAAGACTCATCTGCA-3’
55nts_T	5’-P/TGCAGATGAGTCTTCGAAGACCGTTTCTTTTGGCGAAGACTCATCTGCACGACTC-3’
55nts_B	5’-P/GAGTCGTGCAGATGAGTCTTCGCCAAAAGAAACGGTCTTCGAAGACTCATCTGCA-3’
